# First automated detection of a cardiac arrest using a commercially available smartwatch: a case report

**DOI:** 10.1016/j.resplu.2026.101247

**Published:** 2026-01-29

**Authors:** Wisse M.F. van den Beuken, Pieter R. Tuinman, Beat Nideröst, Sebastiaan A. Goossen, Hans van Schuppen, Stephan A. Loer, Lothar A. Schwarte, Patrick Schober

**Affiliations:** a Department of Anesthesiology, Amsterdam UMC, Amsterdam, the Netherlands; b Amsterdam UMC, Department of Intensive Care, UMC, the Netherlands; c Amsterdam Cardiovascular Sciences Research Institute, Amsterdam University Medical Centre, Amsterdam, the Netherlands; d Utek B.V., Buurmalsen, the Netherlands; e MiBida, Eindhoven, the Netherlands; f Helicopter Emergency Medical Service Lifeliner 1, Amsterdam, the Netherlands

**Keywords:** Resuscitation, Out-of-hospital cardiac arrest, In-hospital cardiac arrest, Automated cardiac arrest detection, Wearable, Smartwatch, Photoplethysmography

## Abstract

**Background:**

Automated cardiac arrest detection aims to shorten the time between arrest onset and emergency medical services activation, thereby reducing the number of unwitnessed out-of-hospital cardiac arrests (OHCA) and shortening time to treatment in witnessed OHCA. Current arrest detection algorithms are largely developed using simulated or artificially induced cardiac arrest data. To our knowledge, this case report provides the first detailed description of the automated detection of spontaneous, non-procedural, end-of-life cardiac arrest using consumer-grade smartwatch-derived sensor data.

**Case report:**

An 82-year-old patient presented to the emergency department with a severe intracerebral hemorrhage with poor prognosis. Following shared decision-making with the family, palliative management was initiated. The patient was continuously monitored with electrocardiography (ECG), invasive arterial blood pressure, and clinical photoplethysmography (PPG). In addition, a commercial smartwatch was placed on the wrist to collect sensor data during the palliative phase and up to 20 min after confirmed cardiac arrest. The smartwatch PPG data were retrospectively analyzed using a previously described diagnostic algorithm. This preliminary algorithm detects circulatory arrest using the photoplethysmography sensor signals acquired from a commercial smartwatch. The algorithm accurately identified the moment of cardiac arrest in concordance with the clinical reference signals. Informed consent was obtained for this research from a legal representative.

**Conclusion:**

Although this controlled end-of-life setting does not represent the circumstances of an OHCA, this case demonstrates the feasibility of detecting true cardiac arrest using a commercial available smartwatch. Prospective studies in real-world OHCA populations are needed to assess clinical performance and practical applicability.

## Introduction

Out-of-hospital cardiac arrest (OHCA) remains a major cause of mortality worldwide.^1^, ^2^, ^3^ Early recognition and rapid activation of emergency medical services (EMS) are essential components of the chain of survival, yet detection is still highly dependent on the presence of a bystander, who is absent in approximately 50% of OHCA cases.^4^ Automating the detection of cardiac arrest may help overcome this critical limitation. Several research groups are developing algorithms capable of identifying cardiac arrest using data from wearable devices.^5^, ^6^, ^7^, ^8^ Wearables contain a variety of physiological sensors, are increasingly used for continuous monitoring, and therefore hold promise for real-time cardiac arrest detection.^9^

Existing algorithms for cardiac arrest detection have been developed and validated primarily using simulated cardiac arrest data in volunteers, or artificially induced or *peri*-procedural cardiac arrests in patients undergoing cardiac and cardiosurgical procedures.^6^, ^7^, ^10^ To our knowledge, the automated detection of a naturally occurring, non-procedural cardiac arrest has not yet been described in detail in peer-reviewed literature, with previous reports limited to an abstract presentation.^11^ We describe what appears to be the first detailed characterization of a commercially available smartwatch successfully capturing physiological data to detect such a cardiac arrest, demonstrating that wrist-worn wearable technology may be capable of detecting not only simulated or induced events, but also true cardiac arrests in clinical practice.

## Case presentation

An 82-year-old male patient was brought to the Emergency Department (ED) by ambulance after experiencing sudden malaise, diaphoresis, and dizziness. On arrival of the ambulance crew, the patient was noted to be increasingly unresponsive with a Glasgow Coma Scale (GCS) score of E3M6V4. During transport, his condition deteriorated further, with episodes of apnea, snoring respirations, and subsequent unresponsiveness. On arrival at the ED, the patient had a GCS score of E4M6V2 and showed miotic pupils that were unresponsive to light. The patient’s clinical condition continued to deteriorate, with the development of bradycardia and increasing periods of apnea. A CT-scan of the brain revealed a right cerebellar parenchymal hemorrhage with intraventricular extension, mass effect on the brainstem and temporal horns, most likely related to underlying hypertension. In addition, the scan showed signs of early hydrocephalus.

**Fig. 1:**
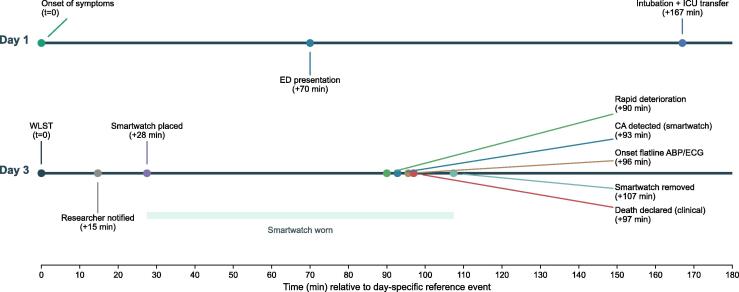
**Timeline**.

Given the poor prognosis of the intracerebral hemorrhage and the patient’s poor clinical condition, and in accordance with the patient’s documented do-not-resuscitate and do-not-ventilate directives recorded by the general practitioner, a shared decision was made with the family to focus on palliative management. Because the patient had explicitly expressed a wish to donate organs, he was transferred to the ICU and intubated solely for the purpose of evaluating potential organ donation. No suitable transplant recipients were identified. After approximately 42 h, the decision was made to withdraw life-sustaining treatment (WLST). The sedation was continued for the comfort of the patient. The patient died approximately 1.5 h after WLST. A timeline of the relevant events is depicted in Fig. 1.

**Fig. 2:**
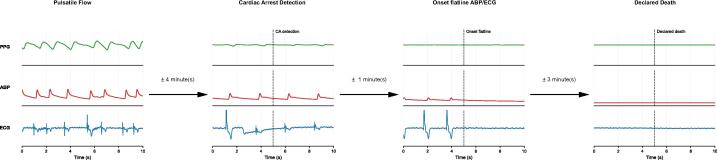
**Cardiac arrest detection**.

As part of a feasibility evaluation, and with approval of the institutional review board and consent from the next-of-kin, a commercial consumer-grade smartwatch, Samsung Watch5 Pro, was placed on the patient’s right wrist approximately 40 min after WLST. The timing of the smartwatch placement on the wrist was based on practical considerations and wishes of the patient’s representative. Fig. 2 includes a representative 10-second segment of physiological signals recorded four minutes prior to cardiac arrest. Smartwatch sensor data were collected using proprietary software developed for the HEART-SAFE project.^5^ Continuous clinical monitoring included electrocardiography (ECG), invasive arterial blood pressure (ABP) monitoring via an arterial catheter in the radial artery, and photoplethysmography (PPG), data were captured and displayed using the Philips IntelliVue MX800 X2 Monitor. These clinical signals served as the reference standard for determining the exact moment of circulatory arrest. At the time the smartwatch was being placed, the patient had atrial fibrillation with a heart rate of 54 beats per minute, an arterial blood pressure of 90/39 mmHg, and a blood oxygen saturation of 34%.

The watch collected sensor data of multiple sensors including green-light PPG, which is typically used for heart rate estimation in smartwatches. We extracted the green PPG signal and retrospectively applied our previously described diagnostic algorithm,^10^ specifically developed to detect circulatory arrest based on PPG waveform characteristics, including peak detection. The algorithm analyses 5 s of green PPG data to determine the presence or absence of cardiac arrest. At the moment the algorithm identified circulatory arrest, the ECG showed agonal bradycardia, with the monitor displaying a heart rate of 0/min. Fig. 2 includes sensor data from the moment of cardiac arrest detection. The patient’s mean arterial blood pressure had fallen to 9 mmHg, with no measurable systolic or diastolic values. However, the arterial blood pressure data showed low-amplitude oscillations, and the hospital monitor’s PPG-derived heart rate still registered 28/min, reflecting residual low-flow agonal cardiac activity. The smartwatch PPG waveform continued to show low-amplitude oscillations at approximately 30 cycles per minute; however, the amplitude was too low to be interpreted as effective pulsatile flow, and the algorithm correctly triggered the cardiac arrest. A rapid decline set in approximately 3 min before the cardiac arrest detection with increasing bradycardia and a gradual decline in blood pressure. No false alarms occurred during the observation period. The smartwatch remained in place until approximately 20 min after confirmed death. Fig. 2 also includes data recorded four minutes after the algorithm detected cardiac arrest and the patient was confirmed dead by a clinician. Supplementary Materials provide a 10-min window of sensor data preceding the clinical confirmation of death.

*This figure displays four representative 10-s segments of physiological signals:*

– *Pulsatile Flow: Recorded four minutes before cardiac arrest, showing normal pulsatile activity.*
– *Onset of Cardiac Arrest: The time point at which the preliminary cardiac arrest detection algorithm identified the onset of cardiac arrest.*
– *Onset flatline ABP/ECG: Time point at which both arterial blood pressure and ECG signals recorded by the Philips monitor become flatlined.*
– *Declared Death: Signals recorded four minutes after cardiac arrest onset, at the time the patient was declared dead by a clinician, demonstrating electrocardiographic asystole and absence of arterial circulation.*

*The y-axis limits are consistent across all panels to facilitate direct comparison. Please note that absolute units are not displayed due to the lack of calibration data; this does not affect the morphology or interpretability of the signals.*

*PPG: Smartwatch photoplethysmography (PPG) signal, normalized and filtered using a 4th order Butterworth bandpass filter (0.5–5 Hz).*

*ABP: Arterial blood pressure (ABP) measured via an intravenous catheter in the a. radialis; ABP data is unfiltered.*

*ECG: Electrocardiogram (ECG), lead II; ECG data is unfiltered.*

*WLST: Withdrawal of life-sustaining treatment*.

## Discussion

In this case report, we demonstrate that a commercially available smartwatch was able to capture sufficient physiological data to correctly identify an actual cardiac arrest using our previously developed detection algorithm. Continuous invasive arterial blood pressure monitoring, ECG, and clinical PPG served as robust reference standards, allowing us to verify the moment of circulatory arrest and compare it to the smartwatch-derived signal. To our knowledge, this is the first detailed documentation in peer-reviewed literature of automated detection of a terminal cardiac arrest following WLST, that was not artificially induced, using data from a commercially available smartwatch.

Although this controlled situation of cardiac arrest occurring in the ICU during palliative withdrawal of treatment does not reflect the full complexity of OHCA, it provides unique insight into the physiological evolution of a cardiac arrest as captured by a consumer wearable. Patients undergoing end-of-life care often experience a progressive decline in cardiac output with bradycardia leading to asystole, rather than a sudden transition into a shockable rhythm (ventricular fibrillation (VF)/ventricular tachycardia (VT)). While OHCA research often focuses on sudden arrhythmic cardiac arrest, a significant proportion of real-world arrests involve gradual physiological deterioration.^12^ In practical implementation, loss-of-pulse detection algorithms will inevitably encounter a range of patient scenarios, including those characterized by the slow-onset phenotype observed in our case. Such gradual deterioration may pose particular challenges for automated algorithms that must determine the precise moment to trigger a cardiac arrest alarm. To detect circulatory arrest, our algorithm utilizes a peak detection mechanism to identify individual pulse waveforms. Crucially, this mechanism incorporates safeguards regarding the minimal amplitude required for a valid beat. As the patient's cardiac output declined, the resulting decrease in pulse amplitude, combined with severe bradycardia, caused the signal to fall below these detection thresholds. Consequently, the algorithm triggered a cardiac arrest alert while minimal residual output was still present, shortly before circulatory arrest was confirmed through clinical monitoring. We consider this timing appropriate, and consider it a true-positive detection even though minimal pulsatile flow was still present, as it identifies hemodynamic collapse and provides a critical window for potential intervention, rather than delaying alert generation until complete circulatory arrest. However, we acknowledge that the intended timing of such alerts involves a fundamental trade-off between early detection to enable timely intervention and the avoidance of false-positive alerts. While the threshold used in this case successfully identified the terminal event, achieving an optimal balance remains a key challenge for the field. The ideal timing for alerting likely varies by clinical context and the specific goals of care, necessitating further prospective investigation to refine these thresholds for broader clinical application.

A key limitation of this report is that the algorithm was applied retrospectively rather than in real time. We opted not to deploy the algorithm live on the device, as it is still under development and we wished to avoid generating alarms that could disturb the patient’s family during the palliative process. Furthermore, this case describes a clinical context characterized by severe hypoxia and terminal circulatory decline, that most likely will differ from the younger and more heterogenous target population for whom automated OHCA detection algorithms are ultimately intended. The profound hypoxic state will most likely not have influenced the signal quality by much, while the light absorption of oxyhemoglobin and deoxyhemoglobin at the green PPG wavelength are similar.^13^, ^14^ This renders light attenuation at that wavelength less sensitive to oxygen saturation changes. Although this case demonstrates technical feasibility of detecting real cardiac arrests, it may not directly reflect clinical performance and practical applicability in real-world settings.

Despite these limitations, this case represents an important step towards bridging the gap between simulated or artificially induced cardiac arrest datasets and true cardiac arrest events. It suggests that wrist-worn wearables could provide sufficient signal quality to support automated cardiac arrest detection algorithms, even in the context of terminal circulatory decline. Future work should focus on prospective, real-time evaluations, including validation in uncontrolled environments where motion artifacts, variable wearing patterns, and diverse cardiac arrest mechanisms may affect performance. This validation is essential before widespread clinical implementation can be considered. Large-scale studies are needed to assess the generalizability and robustness of automated cardiac arrest detection using smartwatches across diverse populations and real-world settings. These studies should evaluate algorithm performance and examine integration into existing emergency response systems. Furthermore, collaboration with regulatory bodies will be necessary to ensure safety, efficacy, and compliance with medical device regulations. Only through rigorous validation and active stakeholder engagement can the potential of smartwatch-based cardiac arrest detection be fully realized and translated into improved patient outcomes. To enhance user acceptance and implementation, and to maximize real-world impact, future studies should also address user-centered concerns, such as privacy, data protection, and accessibility.^15^

## Conclusion

This case-report demonstrated that automated detection of a spontaneous, non-procedural terminal cardiac arrest was possible using the sensor signals of a commercially available smartwatch. Future research should focus on prospective, real-time evaluations in larger cohorts, including out-of-hospital cardiac arrests, to assess real-world performance.

## Informed consent statement

Written informed consent was obtained from a legal representative of the patient.

## CRediT authorship contribution statement

**Wisse M.F. van den Beuken:** Writing – original draft, Visualization, Methodology, Investigation, Data curation. **Pieter R. Tuinman:** Writing – review & editing, Supervision, Investigation, Conceptualization. **Beat Nideröst:** Writing – review & editing, Software, Methodology, Data curation, Conceptualization. **Sebastiaan A. Goossen:** Writing – review & editing, Software, Data curation. **Hans van Schuppen:** . **Stephan A. Loer:** Writing – review & editing, Investigation, Conceptualization. **Lothar A. Schwarte:** Writing – review & editing, Methodology, Investigation, Conceptualization. **Patrick Schober:** Writing – review & editing, Supervision, Project administration, Methodology, Funding acquisition, Conceptualization.

## Declaration of competing interest

The authors declare the following financial interests/personal relationships which may be considered as potential competing interests: The authors declare the following financial interests/personal relationships which may be considered as potential competing interests: Hans van Schuppen reports grants to his institution from the Zoll Foundation and Stryker Emergency Care, both outside the submitted work. Patrick Schober obtained research funding for the HEART-SAFE project form the Top Consortia for Knowledge and Innovation’s (TKI) office of the Dutch Life Sciences & Health (LSH) Top Sector (Health Holland), and reports a grant to his institution outside the scope of this research from Ambu.
