# Supplementary figures and images for: First automated detection of a cardiac arrest using a commercially available smartwatch: a case report
Source: Resusc Plus. 2026 Jan 29;28:101247. doi: 10.1016/j.resplu.2026.101247 (PMC12914423; doi:10.1016/j.resplu.2026.101247)

10-Minute Window (Continuous)

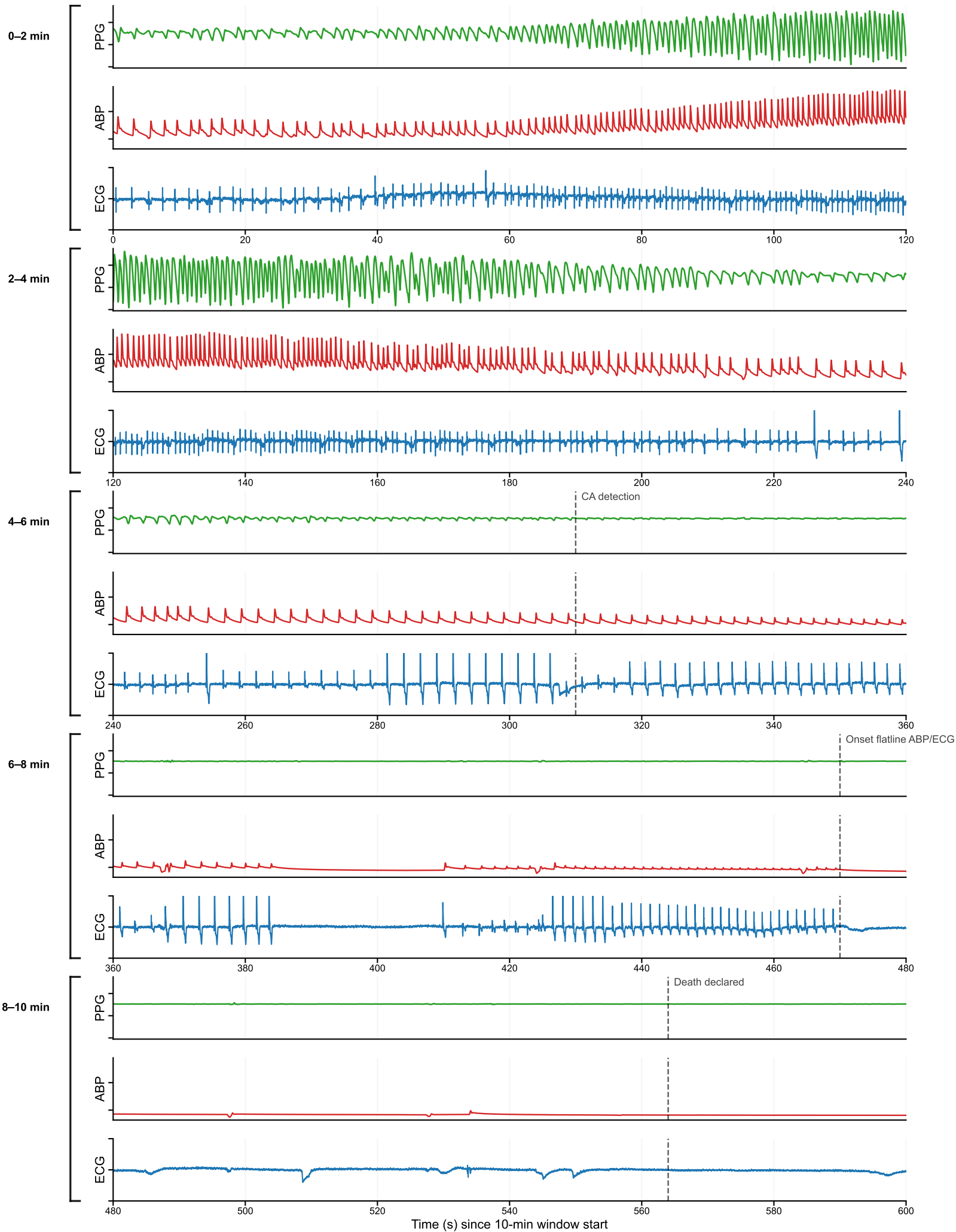

Supplement: Supplementary Data 1 [file mmc1.pdf]
